# Expression of the *Arabidopsis thaliana BBX32* Gene in Soybean Increases Grain Yield

**DOI:** 10.1371/journal.pone.0030717

**Published:** 2012-02-17

**Authors:** Sasha B. Preuss, Robert Meister, Qingzhang Xu, Carl P. Urwin, Federico A. Tripodi, Steven E. Screen, Veena S. Anil, Shuquan Zhu, James A. Morrell, Grace Liu, Oliver J. Ratcliffe, T. Lynne Reuber, Rajnish Khanna, Barry S. Goldman, Erin Bell, Todd E. Ziegler, Amanda L. McClerren, Thomas G. Ruff, Marie E. Petracek

**Affiliations:** 1 Monsanto Company, St. Louis, Missouri, United States of America; 2 Monsanto Research Centre, Monsanto Company, Hebbal, Bangalore, India; 3 Mendel Biotechnology, Inc., Hayward, California, United States of America; USDA-ARS, United States of America

## Abstract

Crop yield is a highly complex quantitative trait. Historically, successful breeding for improved grain yield has led to crop plants with improved source capacity, altered plant architecture, and increased resistance to abiotic and biotic stresses. To date, transgenic approaches towards improving crop grain yield have primarily focused on protecting plants from herbicide, insects, or disease. In contrast, we have focused on identifying genes that, when expressed in soybean, improve the intrinsic ability of the plant to yield more. Through the large scale screening of candidate genes in transgenic soybean, we identified an *Arabidopsis thaliana* B-box domain gene (*AtBBX32*) that significantly increases soybean grain yield year after year in multiple transgenic events in multi-location field trials. In order to understand the underlying physiological changes that are associated with increased yield in transgenic soybean, we examined phenotypic differences in two *AtBBX32*-expressing lines and found increases in plant height and node, flower, pod, and seed number. We propose that these phenotypic changes are likely the result of changes in the timing of reproductive development in transgenic soybean that lead to the increased duration of the pod and seed development period. Consistent with the role of *BBX32* in *A. thaliana* in regulating light signaling, we show that the constitutive expression of *AtBBX32* in soybean alters the abundance of a subset of gene transcripts in the early morning hours. In particular, *AtBBX32* alters transcript levels of the soybean clock genes *GmTOC1* and LHY-CCA1-like2 (*GmLCL2*). We propose that through the expression of *AtBBX32* and modulation of the abundance of circadian clock genes during the transition from dark to light, the timing of critical phases of reproductive development are altered. These findings demonstrate a specific role for *AtBBX32* in modulating soybean development, and demonstrate the validity of expressing single genes in crops to deliver increased agricultural productivity.

## Introduction

The world-wide requirement for grain is predicted to rise seventy percent by the year 2050 [1]. The rise is driven by expanding worldwide population as well an escalating demand for higher protein diets that accompanies growing per-capita incomes [1], [2]. Because the majority of high-quality farm land is already in use for agricultural production, the increasing demand for food and feed necessitates increasing productivity per hectare while conserving natural resources [3]. Historically, gains in agricultural productivity offer both a mechanism to increase agricultural output while simultaneously lessening the impact on land and biodiversity [4], [5]. From 1971 to 2007, crop yields increased from 2 to 2.6 percent annually while the amount of land used in agriculture increased by 0.3 percent per year [6]. While overall agricultural productivity increased in the preceding decades, the productivity gains of soybeans have lagged behind some other major agronomic crops, particularly when compared to maize [7]. Although the commercialization of transgenic crops with both herbicide and insect resistance has led to yield gains through the protection of crop yield [8], there has so far been no introduction of a transgenic crop designed to specifically increase grain yield.

With the aim of developing higher yielding plants, we have pursued a program of screening hundreds of transgenes introduced into soybean. We have conducted multi-location, multi-year field trials with the candidate genes, and have identified genes which lead to yield improvement from these trials. This paper describes the identification of one such yield gene, *Arabidopsis thaliana BBX32*. *AtBBX32* is a member of the B-box gene family and has been implicated in regulating light signal transduction in *A. thaliana* [9]. In this paper we demonstrate that expression of the *AtBBX32* gene in soybean results in increased grain yield per unit area compared to a non-transgenic control of the same genetic background. Additionally, we observed increases in key yield components such as pod number, seed number, and individual seed weight per plant, which are likely the result of increases in the duration of the pod and seed development window in *AtBBX32*-expressing soybean. Furthermore, we observed that *AtBBX32* expression in soybean results in modulation of gene expression during the transition from dark to light, including subtle alteration in the abundance of circadian clock components.

## Results

### *AtBBX32* expression increases soybean yield

Data from *A. thaliana* indicated that overexpression of *AtBBX32* caused increased hypocotyl growth [9], suggesting that, when expressed in a crop plant, the gene might lead to higher overall rates of growth. These results led us to test the efficacy of *AtBBX32* in improving soybean yield. We generated eight independently transformed *AtBBX32* expressing soybean lines and assayed yield in multi-location field trials conducted over three seasons; two seasons in the United States and one season in Argentina. Six of the eight transgenic events showed consistent yield gains (an increase in kilograms of seed per hectare) in a meta-analysis across the three seasons (Table 1). Four of the eight transgenic events yielded more than 5 percent over controls. Transcript analysis from V3 leaf tissue revealed that seven of the eight lines express *AtBBX32* at similar levels, while line 4 does not express the transgene at detectable levels (Table S1).

**Table 1:** *AtBBX32* transgenic soybean plants demonstrate improved grain yield over non-transgenic controls.

	Season 1United StatesN = 10		Season 2United StatesN = 19		Season 3ArgentinaN = 14		Meta-analysis across seasonsN = 43			
Line	Yield(kg/h)	% change vs control	Yield(kg/h)	% change vs control	Yield(kg/h)	% change vs control	Yield(kg/h)	% change vs control	ΔDOF	Δ MAT
1	4725	3.2	3968	8.5**	3766	7.7**	4068	6.9**	0	1.6**
2	4707	3.7	4040	7.2**	3661	3.1	4076	5.3**	−0.4	1.4**
3	4604	−1	3953	6.1**	3481	4.4	3966	4.1**	1.0**	1.8**
4	4277	−6.4**	3777	1.8	3287	−7.3**	3762	−2.3	0.4	0
5	4693	0.3	3972	7.1**	3655	6.4*	4040	5.6**	−0.2	1.3**
6	4814	0.1	3957	8.7**	3519	1.4	4014	4.8**	−0.2	0.9**
7	4491	−4.8*	3867	4.4*	3550	2.4	3917	1.9	−0.7**	0.2
8	4731	5.3*	3902	5.8**	3696	6.5*	4019	5.9**	−0.5	1.0**

Mean yield values (kilograms per hectare) and percent improvement over controls for transgenic plots are shown for three growing seasons. The difference in the day of flowering (DOF) between the transgenic lines and control was calculated to determine delta DOF. The difference in day of final maturity (MAT) was examined in transgenic lines and compared to control to determine delta MAT (units = days). The low yielding event 4 produced no detectable transcript. N represents the number of environments tested. p-values were based on the difference between the transgenic lines and wildtype control.

*p≤0.05,

**p≤0.01.

In addition to comparing grain yield in transgenic events expressing *AtBBX32*, we also characterized two agronomic traits, day-of-flowering (DOF) and maturity (MAT). DOF is defined as the day at which 50 percent of the plants in the plot are in full bloom (R2 stage). Maturity is defined by the presence of 95 percent mature brown pods across the plot (R8 stage). An across season meta-analysis indicated little to no change in day of flowering (with the exception of lines 3 and 7, where the effect was positive in line 3 and negative in line 7). In contrast, we observed an increase in the number of days (1–2 days) to reach final maturity in six of the eight transgenic lines compared to control. To test whether the yield gain was correlated with the maturity delay we collected grain yield data from nine commercially available varieties (Table S2), ranging from relative maturity group 2.7 to 4.4, grown at three of the locations tested in season 1. In these trials we found little to no correlation of yield with maturity, with R^2^ values of 0.07, 0.05, and 0.09 in each of the trials, respectively. These findings agree with previous studies demonstrating a lack of strict correlation of grain yield with the date of final maturity [10]–[11].

### *AtBBX32* impacts key yield component parameters

To understand the physiological impact of *AtBBX32* expression in soybean, we grew two representative *AtBBX32* expressing soybean lines in both controlled environment conditions and in the field and measured the effects of transgene expression on plant growth. *AtBBX32* expression in soybean led to changes in node number, flower number, pod number, seed number, and 100 seed weight, all of which have a clear association with yield [12]. We also found changes in plant height. In growth chamber experiments, the transgenic lines (numbers 1 and 2 from Table 1) showed statistically significant increases in all of the six characteristics measured (Table 2). *AtBBX32* transgenic soybean plants developed 8–10 more nodes, 77–87 more flowers, and 15–17 more pods than did the control plants. The primary yield components, seed number and seed weight, were also positively impacted. AtBBX32 expression led to approximately 23 percent increases in the total seed number of both lines compared to control, while we observed a more modest increase (7 percent) in 100 seed weight in line 2. Plant height also increased from an average of 81 centimeters (cm) in control plants to over 120 cm in both transgenic events. Altogether the data suggest that, under the specific growth chamber conditions tested, *AtBBX32* expression significantly increases component traits associated with yield improvement and plant growth.

**Table 2:** *AtBBX32* transgenic soybean plants increase yield components.

			Field (56 plants m^−2^)				Growth Chamber											
	Yield (kg/h)	% change yield	Height (cm)	% change height	# pods per 0.3 m	% change pods	Height (cm)	% change height	# nodes	% change nodes	# flowers	% change flowers	# pods	% change pods	# seed	% change seed	100 seed weight (g)	% change weight
Control	3632	-	93.5	-	258.3	-	80.6	-	43	-	188.1	-	76.1	-	175.1	-	15.2	-
Line 1	4042	11.3	97.7	4.5	283.9	9.9	121.7	51	52.7	22.6	264.6	40.7	91.2	19.8	218.3	24.7	15.6	2.9^ns^
Line 2	4150	14.2	100.7	7.7	278.4	7.8	122.4	51.8	51.6	20	274.7	46	93.5	22.9	215.3	23.0	16.3	7.0

Both field and growth chamber grown plants show increases in plant height and pod number in the transgenic lines relative to controls. Node number, flower number, seed number, and 100 seed weight were also increased in growth chamber grown plants. Growth chamber experiments were performed in a 10:14 hour photoperiod (Light∶Dark) with 900 mE of light. Data was collected from ten plant replicates that were randomized among entries in the chamber. Field grown plants were grown under standard agronomic conditions and ambient light. All differences between the transgenic lines and control are significant to p<0.05 unless otherwise indicated as (ns) not significant.

Analogous with the increases in yield components observed in the growth chamber study and consistent with results obtained in the multi-location yield trials (Table 1), a single location field trial designed to capture plant phenotypes confirmed that *AtBBX32* expression positively impacted key yield components under field conditions (Table 2). It should be noted that soybean plant architecture varies considerably in response to the different environmental inputs throughout a field growing season. For example, both the number of pods [13] and the extent of branching [14]–[15] are significantly affected by variations in plant density. To minimize differences in plant architecture caused by variable spacing, we over-seeded field soybean plots and thinned to a standard agronomic density (56 plants m^−2^). We measured plant height and found a statistically significant increase, though less dramatic than the increase we observed in the controlled environment study. Additionally we documented a significant increase in pod number from a subsample of plants harvested from a 0.3 m section at the center of the plot (Table 2). These phenotypes, consistent between the growth chamber and field trials, point toward a mechanism by which *AtBBX32* expression in soybean leads to increases in yield components, in turn leading to the increase in grain yield observed in multi-location field trials.

### AtBBX32 extends the duration of pod and seed development in soybean and delays leaf senescence

**Figure 1:**
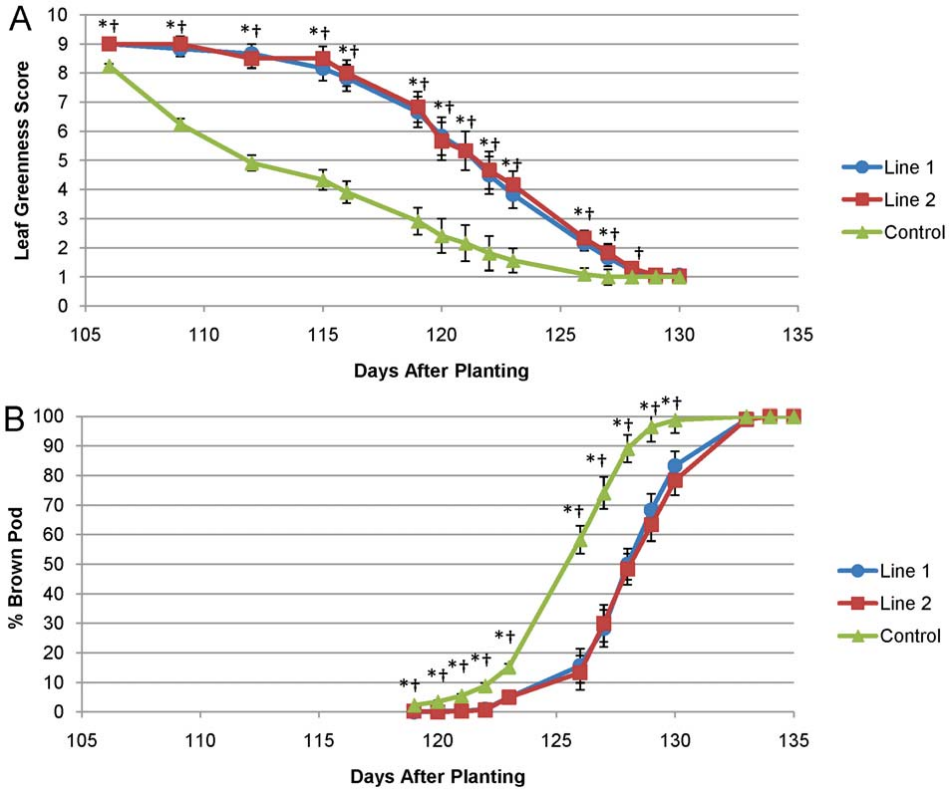
*AtBBX32* expression in soybean delays leaf senescence and brown pod maturity. A) Field grown soybeans were visually assessed and scored every few days late in the season on a whole plot basis according to green leaf color. Leaf senescence was rated on a 1–9 scale based on whole plot appearance. 9 = dark green, no yellow leaves on the top canopy; 5 = 40 percent change yellow leaves, 10 percent change fallen leaves; 1 = more than 95 percent change fallen leaves. B) The same soybean plots were visually inspected for the appearance of brown pods and the percentage of the plot containing brown pods was determined. * Event 1 significantly different from control at p<0.05, † Event 2 significantly different from control at p≤0.05.

Based on the phenotypic observations of increased nodes, flowers, pods, seed, plant height and delayed maturity, we hypothesized that expression of *AtBBX32* in soybean may affect the timing of reproductive development. In order to understand the effect of *AtBBX32* on the timing and duration of soybean development, we measured the number of days to reach specific growth stages in two *AtBBX32*-expressing lines (event 1 and 2 from Table 1) and control [16]. Both *AtBBX32* transgenic events initiated flowering (R1 stage) and pod development (R3 stage) at the same time as control (Table 3). However, the number of days to reach beginning maturity (R7) was significantly increased in both events, suggesting that the duration of pod and seed development (R3-R7) is increased in *AtBBX32*-expressing transgenic events by approximately three to four days (Table 3). In addition to the analysis of developmental timing, we assessed the number of days to reach 95 percent leaf senescence by using a visual assessment scale of 1 to 9 to score soybean plots based on the degree of leaf greenness and percent fallen. Results indicate that *AtBBX32* expressing soybeans maintain a nearly full complement of green leaves approximately ten days longer than control (Figure 1), thus the initiation of senescence is delayed. The rate of leaf senescence increases near the end of the senescence window in transgenic events such that both events and control reach 95 percent fallen leaves approximately four days apart. Thus, the increase in the number of days to reach beginning of maturity (R7) observed in both transgenic lines is accompanied by a stay green phenotype, which we propose may help to provide resources to maintain extended reproductive development. In addition to leaf senescence, we also tracked the timing of plants to reach final maturity (95 percent brown pod formation). Similar to the leaf phenotype and consistent with our analysis of developmental timing, we found that final maturity, or 95 percent brown pod formation, was delayed in both lines by approximately three days relative to control. This final delay in maturity is likely due to the developmental delay observed between the R3 and R7 stages (Figure 1, Table 3). While the three day delay in maturity was larger than that observed in the field trials reported in Table 1, the difference is likely a result of the inherent variability in developmental timing of field grown plants and the fact that yield trials in Table 1 were multi-year and multi-location, while the phenotypic analysis above was conducted in a single year, single location trial.

**Table 3:** *AtBBX32* extends the reproductive period between R3 and R7 developmental stages in soybean resulting in a delay in final maturity compared to control.

	Developmental Stage			
	R1	R3	R7	R8
Control	38.1	57.8	112.5	120.4
Event 1	39.3	57.7	115.8*	122.8*
Event 2	39	57.2	116.7*	123.6*

The timing of reproductive development was measured according to standard methods [16] in ten field plot replicates for each line. R1 is the initiation of flowering. R3 is the onset of pod development. R7 is the beginning of maturation. R8 is the stage where 95 percent of the pods are physiologically mature. The number of days to reach each developmental stage was calculated on a whole plot basis and the mean is indicated below, where units are days after planting.

*p<0.05.

### Microarray analysis of *AtBBX32* expressing soybean lines reveals subtle alteration of gene expression near dawn

**Figure 2:**
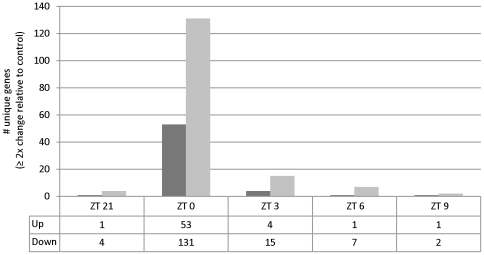
Microarray data from field grown plants. Microarrays performed on tissue sampled throughout the day from two *AtBBX32*-expressing lines (lines 1 and 2 from Table 1) in the field demonstrate 219 genes show 2–8 fold changes (8-fold is maximum change observed) in abundance in both transgenic events relative to the control and that the majority of these changes occur around ZT 0 (6 am). Dark bar represents genes increased in abundance and light bar represents genes decreased in abundance. All changes significant at a false discovery rate of 5 percent.

Results from the overexpression of *BBX32* in *A. thaliana* indicate that *BBX32* acts to modulate the expression of light-regulated genes during the transition from dark to light [9]. In order to test how the expression of *AtBBX32* affected the regulation of gene expression in soybean under agronomically relevant conditions, we performed a microarray analysis (Genbank accession GSE30828) on field grown plants from lines 1 and 2 (Table 1) sampled at five timepoints around dawn; ZT 21 (3 am), ZT 0 (6 am), ZT 3 (9 am), ZT 6 (12 pm), and ZT 9 (3 pm). We found that the expression of *AtBBX32* in soybean affects the abundance of specific gene transcripts and that the majority of these changes in gene expression occur at dawn (ZT 0) (Figure 2). Of the 219 unique genes that showed significant changes of 2 to 8-fold (maximum fold change observed) in transcript abundance at any of the five timepoints sampled, 84 percent of those genes were altered at dawn (ZT 0). Thus, although *AtBBX32* is constitutively expressed in soybean, the microarray data indicate that the influence of *AtBBX32* on the expression of other genes is subtle and restricted to dawn, suggesting that *AtBBX32* functions within an existing framework of gene regulation in soybean.

### Soybean contains B-box genes that are functionally similar to the Arabidopsis *AtBBX32* gene

Since constitutive expression of the *A. thaliana* B-box gene *BBX32* leads to changes in soybean gene expression that are restricted to a specific time of day, we suspected that soybean may encode homologs of *AtBBX32* with similar function. In order to better understand the existing pathway in soybean that *AtBBX32* functions in and identify other candidate genes that lead to increased yield when overexpressed in soybean, we identified the B-box family in soybean, including the soybean homologs of *AtBBX32*. We generated a phylogenetic analysis of the soybean and *A. thaliana* B-box gene families and further analyzed the expression patterns of the soybean B-box gene family in the microarray described above. The *A. thaliana* genome encodes 32 predicted B-box domain proteins [17], while the paleopolyploid soybean genome encodes 61 predicted B-box containing proteins (Figure S1, Table S3). Microarray analysis from R5 stage field grown wildtype soybean plants indicated that a significant proportion of the soy B-box genes are temporally regulated. Phylogenetic analysis of the soybean B-box family was used to identify putative soybean homologs of *AtBBX32*. Of the *AtBBX32*-like soybean genes that encode a single B-box domain, thirteen grouped with the seven Arabidopsis single B-box genes (Figure S2). Of these, *Glycine max BBX52 (GmBBX52)* and *Glycine max BBX53 (GmBBX53)* have the closest phylogenetic relationship to the *A. thaliana BBX32* gene and show evidence for a microsyntenic relationship between the Arabidopsis and soybean genomic regions [18]. The two soybean genes are similar in both nucleotide (92 percent identical) and amino acid sequence (88 percent identical) and presumably arose from the duplication of the soybean genome. Like *AtBBX32*, both soybean genes are predicted to encode a single N-terminal B-box domain. Additionally, both soybean genes showed temporal patterns of transcript abundance with peaks in expression at similar times to each other in the microarray experiment (Figure S1), though each are detected by different probesets (Table S3).

To examine whether *GmBBX52* and *GmBBX53* are functional homologs of *AtBBX32*, we tested whether overexpression of each gene can produce phenotypes in soybean similar to those caused by expression of *AtBBX32*. To do this, we generated transgenic lines that constitutively express either *GmBBX52 or GmBBX53* as well as lines containing a miRNA construct designed to decrease transcript levels of *GmBBX52*. We tested eight independently generated transgenic lines of both the *GmBBX52* constitutive expression construct and the *GmBBX52*- miRNA construct and 4 independent events from the *GmBBX53* expression construct in a single year of field trials designed to assess yield (Table 4). Lines overexpressing *GmBBX52* yielded, on average, 6.1 percent more kilograms per hectare than did wildtype control plants, while the top performing line yielded 9.0 percent more per hectare. Transgenic lines overexpressing *GmBBX53* yielded 4.1 percent higher than the wildtype control, while the top event improved yield by 6.7 percent over the wildtype control. In contrast, miRNA mediated suppression of the *GmBBX52* transcript led to a significant decrease in yield (Table 4). The *GmBBX52* miRNA lines yielded, on average, 5.5 percent less than control lines while the lowest yielding line across the eight miRNA lines produced 11.8 percent less than controls. Confirming the miRNA impact, seven of eight events showed significant decreases in *GmBBX52* transcript levels (Table S1). These data indicate that overexpression of the soybean homologs of *AtBBX32*, *GmBBX52* and *GmBBX53*, enhances yield similarly to what was observed in soybean plants expressing the *A. thaliana* gene, *BBX32*. In addition, we found that overexpression of either *GmBBX52* or *GmBBX53* delayed maturity by 2–4.5 days compared to control, while the day of flowering compared to control was not consistently affected (Table 4). Neither day of maturity or day of flowering was consistently altered in soybean expressing the *GmBBX52* miRNA construct. These data support the hypothesis that *GmBBX52* and *GmBBX53* share a comparable mechanism of action to *AtBBX32*. Furthermore, the field data from our RNAi lines suggest that wild-type expression levels of the *GmBBX52* gene may be required to maintain yield potential in soybean. Our findings suggest that the soybean and *A. thaliana* genes likely perform similar *in-vivo* functions and that the *AtBBX32* gene product in soybean is likely to function within the same biochemical framework as the endogenous soybean homologs of *AtBBX32, GmBBX52 and GmBBX53*.

**Table 4:** Overexpression and suppression of *GmBBX*52 and *GmBBX*53 alters yield.

Construct	Event	Yield (kg/h)	% change vs control	Δ DOF	Δ MAT
*GmBBX52* overexpression	1	5092	3.3	1.9**	4.7**
*GmBBX52* overexpression	2	5170	4.9*	0.1	3.8**
*GmBBX52* overexpression	3	5304	7.7**	−2.7**	3.5**
*GmBBX52* overexpression	4	5154	4.6*	−0.3	3.7**
*GmBBX52* overexpression	5	5280	7.2**	−1.7**	2.4**
*GmBBX52* overexpression	6	5307	7.7**	0.4	3.1**
*GmBBX52* overexpression	7	5368	9.0**	−1.4**	3.6**
*GmBBX52* overexpression	8	5131	4.1	−0.6	3.9**
*GmBBX53* overexpression	1	5023	6.7**	−0.3	2.4**
*GmBBX53* overexpression	2	4939	4.9**	−0.5	1.2*
*GmBBX53* overexpression	3	4806	2.1**	0.3	3.9**
*GmBBX53* overexpression	4	4844	2.9**	0.02	1.3*
*GmBBX52* miRNA	1	4393	−11.8**	0.4	−1.9**
*GmBBX52* miRNA	2	4725	−5.1**	1.1**	0.1
*GmBBX52* miRNA	3	4757	−4.5*	1.1**	0.4
*GmBBX52* miRNA	4	4807	−3.4	−0.3	0.4
*GmBBX52* miRNA	5	4674	−6.1**	2.8**	2.0**
*GmBBX52* miRNA	6	4910	−1.4	−0.8	0.1
*GmBBX52* miRNA	7	4812	−3.3	0.2	−0.04
*GmBBX52* miRNA	8	4556	−8.5**	−0.3	−0.1

Analysis of individual lines from one season of field testing of soybean plants overexpressing the soybean homologs of *AtBBX32*, *GmBBX52* and *GmBBX53*, demonstrate that the expression of the soybean genes leads to significant yield improvements relative to control. miRNA mediated knockdown of *GmBBX52* leads to decreased yield relative to control. The difference in the days to flower (DOF) between the transgenic lines and control was calculated to determine delta DOF. The difference in days to final maturity (MAT) was examined in transgenic lines and compared to control to determine delta MAT (units = days). p-values were based on the difference between the transgenic lines and wildtype control.

**p≤0.05,

*p≤0.1.

### *AtBBX32* expression in soybean affects the regulation of clock related genes

**Figure 3:**
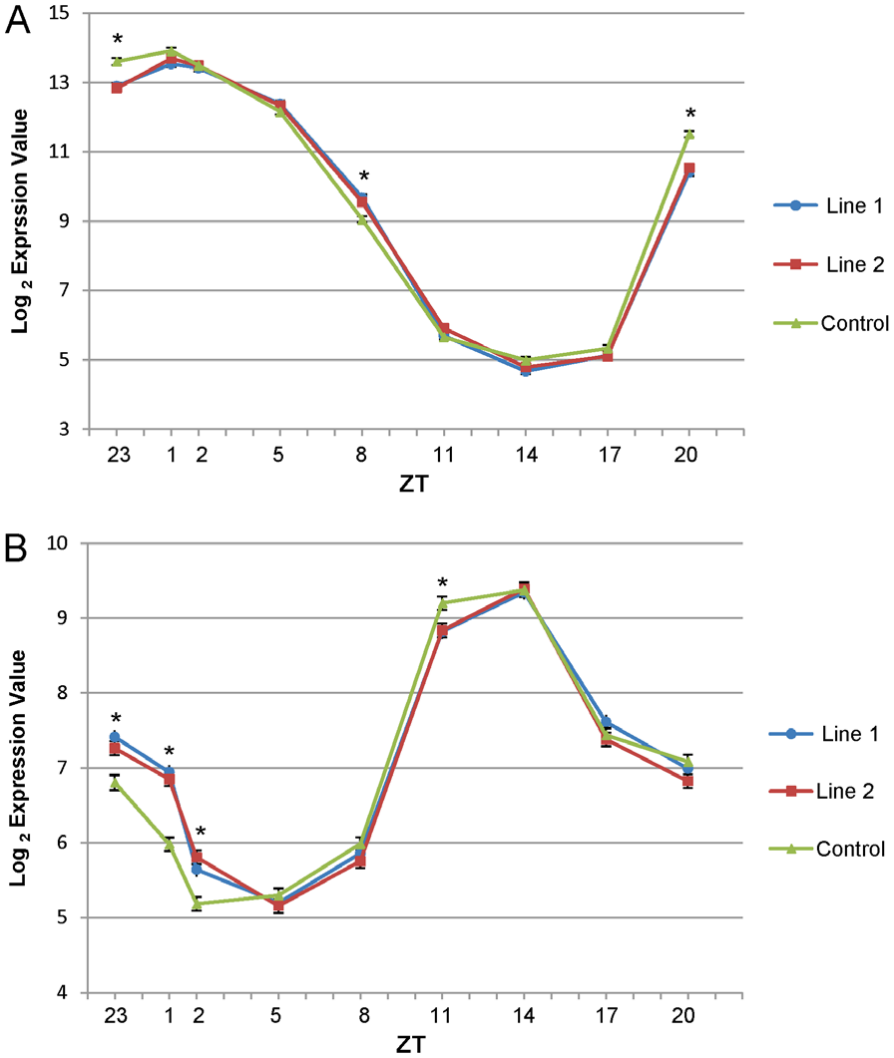
Expression of *AtBBX32* in soybean affects the transcript abundance of central clock components near ZT 0. Levels of both central clock components *GmLCL2* (A) and *GmTOC1* (B) were assayed by quantigene RNA extraction and expression analysis from V2 leaf tissue harvested from soybean plants grown in a controlled environment. Growth chamber experiment was performed in a 14:10 hour photoperiod (Light∶Dark) with 650 mE of light. p-values based on the difference between both transgenic lines and wildtype control. * p≤0.05. Where error bars are not visible they are smaller than the data points.

The microarray data suggested that the majority of genes affected by the expression of *AtBBX32* in soybean are affected during the transition from night to day and are themselves temporally expressed in wildtype soybean, suggesting that they may be diurnally regulated. Because the microarray experiments did not address the entire 24 hour cycle, and since the circadian clock is known to regulate downstream processes such as plant growth and development in response to environmental inputs such as light in *A. thaliana* [19], [20], we sought to further investigate the effects of *AtBBX32* expression in soybean on core components of the circadian clock, LHY/CCA1 and TOC1, over a 24 hour time course. In wildtype soybean the homologs of the core clock genes, LHY-CCA1-like2 (*GmLCL2*) and *GmTOC1* [21], show peak expression in the late-morning and in the evening respectively (Figure 3), similar to peak expression times previously observed in Arabidopsis [22], [23]. In both the microarray (Table S4) and targeted analysis (Figure 3) we found that the level of expression (but not the timing of peak expression) of both of these central clock components is altered in two transgenic soybean lines grown in a controlled environment. The transcript abundance of *GmLCL2* was significantly reduced in both transgenic lines compared to the control at ZT 20 and ZT 23, while the abundance was increased in both lines compared to control at ZT 8 (Figure 3). The largest effects, while still subtle, occurred in the four hours prior to the onset of light. The transcript abundance of *GmTOC1* was oppositely affected, demonstrating increased abundance at ZT 23, ZT 1, and ZT 2 and decreased abundance at ZT 11 in both events compared to control (Figure 3). The observed effects on GmTOC1 expression were also subtle and more evident near the onset of light. In addition, we examined the expression profiles of other genes commonly associated with the plant circadian clock [24], including CO, PRR7/9 and GI, and identified very few significant changes in their expression profiles by microarray (Table S4). We were not able to identify probesets that specifically target homologs of ELF3/4 or LUX in soybean.

To further test whether the soybean homologs of *AtBBX32*, *GmBBX52* and *GmBBX53*, are functionally analogous to *AtBBX32*, we measured the transcript abundance of *GmTOC1* and *GmLCL2* in two lines overexpressing either *GmBBX52* (line 7) or *GmBBX53* (line 2) from previous yield tests over a 24 hour time course (Figure S3). In both lines we observed similar results to those in *AtBBX32* expressing soybean lines; *GmLCL2Y* expression is reduced near ZT 0, while *GmTOC1* expression is increased near ZT 0 in transgenic lines compared to control. In addition to the yield data, these data further support our hypothesis that the two soybean homologs have a similar role to *AtBBX32*.

## Discussion

We demonstrate that the expression of *AtBBX32* in soybean leads to year after year improvements in yield across multiple field seasons. Our observations suggest that the yield gain is a consequence of physiological changes in the plant that lead to increased node number, flower number, pod number, and finally seed weight and number. Furthermore, we demonstrate that *AtBBX32* expression leads to changes in the duration of the reproductive developmental stages between R3 (onset of pod development) through R7 (beginning maturity). Changes in the abundance of clock gene transcripts during the transition from dark to light suggest that *AtBBX32* dependent changes in plant development may be in part a consequence of the modulation of expression of circadian clock components by *AtBBX32*.

While it is significant that the observed phenotypic changes were induced by the expression of a transgene, the changes observed are consistent with the established understanding of physiological changes associated with yield gain in soybean. Yield improvement in soybean results from increased seed number per area [25]. Increased seed number per area is in turn driven in part by the amount of assimilate captured by plants between the R1 through R7 [12] stages of growth. Experiments where soybean plants were defoliated at different developmental times demonstrate that the R4 through late R5 stages are the most critical for soybean yield [26] and that removal of leaves during these stages has the greatest impact on yield. The observation that *AtBBX32* expression in soybean increases the duration of the R3 through R7 stages is well aligned with the biological understanding of the relationship between developmental timing and yield in soybeans [27]. Thus in this study, as in previous studies [10], [11], we found that increased yield was correlated with the length of the grainfill (R3-R7) period rather than with the timing of final maturity (R8).

How does AtBBX32 expression lead to differences in the duration of reproductive development? The molecular mechanisms underpinning the onset of the transition from vegetative to reproductive development are relatively well understood in Arabidopsis and are conserved in other plant species where they have been investigated [28], [29]. In Arabidopsis the switch from vegetative to reproductive development is regulated largely through the integration of light and clock regulated pathways. Under long day conditions, the clock regulated protein CONSTANS (CO) is stabilized [30] and triggers the expression of the floral initiation gene FT [31]. Mis-expression or mutation of photoreceptors such as PhyA [32], circadian clock components CCA1 [33]–[34], or the ubiquitin ligase component COP1 [35] lead to alterations in the initiation of floral development. While marked changes in the actual initiation of reproductive development were not observed in soybean expressing *AtBBX32*, the alteration in the timing of later stages of development suggest a role for *AtBBX32* in regulating the duration of reproductive developmental phases post the initial onset of flowering. We speculate that like CONSTANS, AtBBX32 may play a role at the interface between light and clock to modulate output pathways such as the timing and duration of specific reproductive stages. In *A. thaliana*, *BBX32* represses the activity of the Hy5 transcriptional complex during the transition from dark to light and modulates the expression of circadian clock components such as PRR9 and CCA1 [9]. Our understanding of the role of *AtBBX32* in *A. thaliana* and soybean, demonstrating that *AtBBX32* acts to alter the expression of circadian clock genes specifically at dawn, suggests that *AtBBX32* modifies the input of light to the clock to result in a subtle dampening of clock rhythms near dawn. As a consequence, the duration of R3 to R5 stages, when pod and seed development in soybean occurs, is extended, resulting in increased pod number, seed number, and individual seed weight which contribute to increased grain yield. These findings are directly relevant to soybean agriculture and point towards the possibility of using similar approaches to improve crop plants in general.

## Materials and Methods

### Overexpression and miRNA constructs

*AtBBX32* (AT3G21150) was isolated from *Arabidopsis thaliana* and cloned into an *Agrobacterium tumefaciencs* vector for transformation into soybean according to previously published methods (Martinell et al., 2002). pMON81312 (GenBank JN400384) was generated through the cloning of *AtBBX32* into pMON74532 (GenBank JN400386), downstream of the Cauliflower mosaic virus enhanced 35S (eCaMV 35S) promoter and preceding the 3′ UTR from the *Gossypium barbadense* E6 gene (GenBank GHU30505, nucleotides 965–1142). *GmBBX52* (pMON108080, GenBank JN400381) was cloned into the parent vector pMON100407 (GenBank JN400385). The CaMV35S promoter was followed by the petunia HSP70 5′ UTR (CAGAAAAATTTGCTACATTGTTTCACAAACTTCAAATATTATTCATTTATTTGTCAGCTTTCAAACTCTTTGTTTCTTGTTTGTTGATT). The coding region of *GmBBX52* was followed by the *G. barbadense* 3′ UTR from the E6 gene. *GmBBX53* (pMON98939, GenBank JN400382) was cloned into pMON82053 (GenBank JN400387) and is flanked by the CaMV35S promoter and *G. barbadense* E6 3′ UTR. The artificial microRNA construct (pMON93914, GenBank JN400383) was cloned according to published guidelines (Schwab et al., 2005) and is designed to target CTGAGTGTGTGCCTGGGAAA in *GmBBX52*. The sequence is similar to the corresponding sequence in *GmBBX53* (GGAGGTGTTTGAGAAA) albeit with a single G to A transition at position 12 of the targeted sequence. As a result no significant decrease in *GmBBX53* expression was observed. The DNAs were cloned into a microRNA cassette vector (pMON99036, GenBank JN400388) flanked by the CaMV35S promoter and the *G. barbadense* E6 3′ UTR. The *AtBBX32* expression construct was transformed into Asgrow line A3244, while the *GmBBX52* and *GmBBX53* overexpression and miRNA constructs were transformed into Asgrow line A3525 (Table S2).

### Multi-location Yield Trials

Yield was collected from *AtBBX32* trials (pMON81312) across three seasons. Two row, 4.6 meter plots (0.9 meter alley, 0.75 meter row space) were planted in a group unbalanced block design. Each of the eight *AtBBX32* transgenic soybean events (Table 1) was paired with the non-transgenic, wild-type control and randomly assigned to either one of the two split plots in the whole plot and plots were completely randomized within each replication for each environment. Standard soybean production practice was used in plot management. In 2004 trials were planted at 12 environments in the United States, 2 replications per environment. In 2005 trials were sown at 24 environments in the United States with 3 to 8 replications per environment. In 2005/2006 trials were planted at 16 environments in Argentina with 2 to 8 replications per environment. Trials with a coefficient of variation ≥15 percent or subject to damages by severe environmental factors were dropped from analysis, resulting in 10, 19, and 14 environments analyzed in 2004, 2005, and 2005/06, respectively (Table 1). Line numbers are consistent in tables 1, 2, 3, and S1. For example line 1 in table 1 is the same transgenic event as line 1 in table 2.

Yield was collected from *GmBBX52* and *GmBBX53* overexpression and miRNA constructs from one season. Events overexpressing *GmBBX53* (pMON98939) were evaluated in 2009 in two row, 4.6 meter plots (0.9 meter alley, 0.75 meter row space) planted in a Group Unbalanced Block Design (GUBD). Each of four pMON98939 events were randomly assigned to plots within a dedicated pMON98939 block. Every block (1 per replication) was randomized within each replication for each environment. This trial was planted at 22 environments in the United States with 3 replications per environment. Standard regional agronomic soybean production practices were used for trial management. Environments or replications within an environment with a coefficient of variation ≥15 percent or damaged by severe environmental were excluded from analysis, resulting in 21 environments included in analysis of yield. Day of flowering and day of maturity data were collected on each event from 6 and 8 environments, respectively.

Events overexpressing *GmBBX52* (pMON108080) or miRNA of *GmBBX52/53* (pMON93914) were evaluated in 2009 in two row, 4.6 meter plots (0.9 meter alley, 0.75 meter row space) planted in a GUBD design. Each of 8 events of pMON108080 and pMON93914 were randomly assigned to plots within dedicated pMON108080 and pMON93914 blocks. pMON108080 and pMON93914 blocks were randomized within each environment. In 2009 events of pMON108080 and pMON93914 were planted at 10 environments in the United States with a single replication at each environment. Standard regional agronomic soybean production practices were used for trial management. Environments or replications within an environment with a coefficient of variation ≥15 percent or damaged by severe environmental were excluded from analysis, resulting in 9 and 10 environments included in analysis of yield for pMON108080 and pMON93914, respectively. Day of flowering data were collected from 7 environments for both trials and day of maturity data were collected from 6 and 7 environments for BA831 and BA832, respectively.

Day of flowering data was collected on each plot of each event when 50 percent of plants within the plot were at full bloom. Day of maturity data was collected on each plot of each event with 95 percent of the pods in the plot had turned a brown color.

Across site analysis for *AtBBX32* (pMON81312) was performed according to the group unbalanced block 2 (GUBD2) design for each year of testing. Group block designs (GBD) randomly lay out entry groups within reps, where entry groups are formed by grouping entries based on some attribute, for example, by genetic background. Entry groups can be of equal size (balanced block- GBBD) or unequal size (unbalanced block - GUBD). GUBD2 stands for the GUBD with two factors; for example, entry group and entry. Entries within an entry group remain together in each of the replicates, but are placed in random order within their own group. Entry groups are positioned in the replicates in random order.

For experiments with GUBD2, the statistical model for across locations analysis is
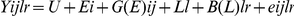
where Y_ijlr_ is the observation for i^th^ entry at the r^th^ rep of l^th^ location, U is the overall mean, E_i_ is the factor 1 effect, G(E)_ij_ is the factor 2 in factor 1 effect, L_l_ is the location effect, B(L)_lr_ is the replication effect within location, and e_ijlr_ is the residual error. E, and G(E) are fixed effects, and L, B(L), and e are random effects and follow normal distributions with mean zero and variance σ^2^_L_, σ^2^_B(L)_, and σ^2^_e_, respectively. Statistical analyses were carried out using PROC MIXED procedure of SAS/STAT of SAS® 9.2.

When data are collected across several sites and years, a meta-analysis is conducted to summarize the effect size. If the entries of interest and their own control entries are pulled out from trials across different environments (combinations of years and sites) with GUBD (Grouped Unbalanced Block Design), for meta-analysis purpose, the study is also considered as coming from a GUBD if the blocking is well maintained. The statistical model for meta-analysis is the same as the GUBD2 above. Statistical analyses were carried out using PROC MIXED procedure of SAS/STAT of SAS® 9.2.

Across site analysis of *GmBBX53* (pMON98939) was performed according to the 3 factor nested design. In the model, construct is the factor 1, event is factor 2 nested within construct, and gene of interest is factor 3 nested within construct and event combination. The analysis used the mixed model:
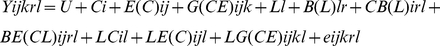
where Y_ijkrl_ = observation on i^th^ construct and j^th^ event and k^th^ gene of interest in the r^th^ rep of l^th^ location, U = overall mean, C_i_ = construct effect, E(C)_ij_ = effect of event within construct, G(CE)_ijk_ = effect of gene of interest within construct and event combination, L_l_ = location effect, B(L)_lr_ = random replication effect within a location, CB(L)_irl_ = construct by random replication within location interaction effect, BE(CL)_ijrl_ = random event within construct by replication within location interaction effect, LC_il_ = random location by construct interaction effect, LE(C)_ijl_ = random location by event within construct interaction effect, LG(CE)_ijkl_ = random location by gene of interest within construct and event combination interaction effect, and e_ijkrl_ = residual error. In the model, C, E(C) and G(CE) are fixed effects and other terms are random effects. Statistical analyses were carried out using PROC MIXED procedure of of SAS/STAT of SAS® 9.2.

Across site analysis of *GmBBX52* (pMON108080) or miRNA of *GmBBX52* (pMON93914) was performed according to the GUBD2 design. The 2 factor GUBD spatial model was fit to the across location analysis. Construct was factor 1 and event within construct was factor 2.

The model for across location analysis is
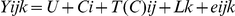
where Y_ijk_ = observation (spatially adjusted value from by-location analysis) from the k^th^ location on the j^th^ event of the i^th^ construct, and U = overall mean, C_i_ = i^th^ construct effect, T(C)_ij_ = effect of j^th^ event nested within i^th^ construct, L_k_ = effect of k^th^ location, e_ijk_ = residual error associated with Y_ijk_. C_i_ and T(C)_ij_ are fixed effects. Distribution of e_ijk_ is assumed to be normal. The variance-covariance matrices of the spatially adjusted values from individual by-location analyses are put together to form a block diagonal matrix that is used as the variance-covariance matrix for the distribution of e_ijk_. Statistical analyses were carried out using PROC MIXED procedure of of SAS/STAT of SAS® 9.2.

Linear regression is an approach to model the relationship between a scalar variable y and one or more variables denoted x or X. In linear regression, data are modeled using linear functions, and model parameters are estimated from the data. In order to understand if there is a linear relationship between yield (YLD) and maturity (MAT), a data set of ten commercial varieties (table S2) tested at 3 locations in Season 1 of *AtBBX32* trials were used. The simple linear regression model between YLD and MAT at individual locations is
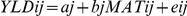
where YLD_ij_ and MAT_ij_ were the observed yield and maturity for variety i at location j, a_j_ is the intercept value (the YLD value when MAT = 0) and b_j_ is the slope (the change in YLD for one unit change in MAT) for location j, and e_ij_ is the residual. A t-test is used to check if the slope is significantly different from 0, indicating no linear relationship between YLD and MAT. R-square (*R* ^2^) is a statistic to measure how well future outcomes are likely to be predicted by this linear model and an *R* ^2^ of 1.0 indicates that the regression line perfectly fits the data.

### Timing of plant development measurements

A field study was conducted at Jerseyville, IL with 10 plot replications per entry. Each plot consisted of four rows 0.75 m apart and 3.6 m long. Plots were over-seeded and thinned at the V2 stage to a final population of 38 plants m^−2^. Soybean developmental stages [16] were determined on an approximate 2-day interval following soybean emergence on 10 consecutive plants.

Statistical analysis was carried out using PROC MIXED procedure of SAS/STAT 9.2. The statistical model for this experiment, which was conducted with group unbalanced block design (GUBD), was based on the observations measured on plot- basis as described below.
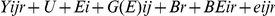
where Y_ijr_ = unique individual observation, U = overall mean, E_i_ = event effect, G(E)_ij_ = gene within event effect, B_r_ = random replication effect, BE_ir_ = random replication by event interaction effect, and e_ijr_ = residual error. E and G(E) are fixed effects, and others are random effects.

### Plant height and Yield Components in Controlled Environment

Walk-in growth chambers were used to grow soybean plants with 10 h–14 h (day-night) photoperiod, a 28–22°C (day-night) temperature, and a fluence during the day of 800 µE of light. Seeds were planted 2.5 cm deep in soil (Metro 350) in a 25.4 cm pot. Pots were inoculated immediately prior to planting by sprinkling 1.25–1.5 g of inoculant (Rhizo Flo granular) mixture around the small hole made for seed placement. Pots were soaked daily for 15 min via sub-irrigation just before the photoperiod began. Nutrient solution (Jack's water soluble fertilizer, 15-5-15, 227 grams/50 liters) was applied three days a week through the sub-irrigation system. Ten plant replicates per entry were included in the study and randomized throughout the growth chamber.

New flowers were counted every day or every other day and recorded electronically in Excel.

Total nodes and total pods were recorded at maturity (R8). At maturity pods were collected from each plant and dried in an oven. Seeds were later removed from each pod and counted in an Old Mill seed counter, Model 850-2. The weight of 100 seed per plant was determined in order to approximate any changes in individual seed weight. Plant height was measured from the soil surface to the apical meristem using a bar coded ruler and a barcode reader (Symbol LS4000i) connected to a Panasonic Toughbook computer.

The experiment was analyzed according to a 2-factor nested design with background as factor 1 and entry as factor 2. A mixed model was used to analyze the data as explained below:
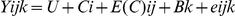
where Y_ijk_ = unique individual observation, U = overall mean, C_i_ = background effect, E(C)_ij_ = entry effect, B_k_ = random block effect, and e_ijk_ = residual error. Statistical analysis was carried out using PROC MIXED procedure of SAS/STAT 9.2.

### Leaf senescence, brown pod maturity and field yield components

A field study was conducted at Jerseyville, IL following a split plot design with three plot replications per entry. Each plot consisted of two rows 0.75 m apart and 3.6 m long. Plots were over-seeded and thinned at the V2 stage to a final population of 56 plants m^−2^. Leaf senescence was rated on a 1–9 scale based on whole plot appearance. 9 = dark green, no yellow leaves on the top canopy; 5 = 40 percent yellow leaves, 10 percent fallen leaves; 1 = more than 95 percent fallen leaves. Assessment was determined based on visual inspection. Maturity was rated on 0—100 percent scale based on the appearance of brown pods across the whole plot based on visual observation. Pod numbers were counted when 80 percent of the pods turned brown across the field. Plants were harvested from a 0.30 m section and the number of pods at each node on the main stem and branches was determined. Plant height was measured at R5 stage by using a barcoded ruler and a barcode scanner connected to a laptop computer. The barcoded ruler was placed in the middle of the row in a plot and the barcode corresponding to the average height of the row was scanned. Yield was determined after combine harvest of the plots used to collect physiology measurements. Data were analyzed with a split-plot design. Thinning density was the whole plot factor and event was the split factor. Individual replicated site analyses used a mixed model
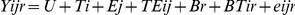
where Y_ijr_ = unique individual observation, U = overall mean, T_i_ = density effect, E_j_ = gene effect, TE_ij_ = density by gene interaction effect, B_r_ = random replication effect, BT_ir_ = random replication by density interaction, and e_ijr_ = residual error. T, E and TE are fixed effects and others are random effects. Statistical analyses were carried out using PROC MIXED procedure of SAS/STAT 9.2.

### Phylogenetic analysis

Soy sequences having a B-box domain were collected from the GIS data base, comprising of gene predictions from Soybean genotype, Williams 82, whole genome sequence assembly. Of the initial ∼100 sequences collected, sequences representing pseudogenes and platz domain containing proteins were removed. In addition, 23 sequences that represented allelic/splice variants were separated and not included in the phylogenetic analysis. A total of 61 sequences representing the soy B-box genes were used to build the phylogenetic tree (Table S3). The soy sequences, along with 32 Arabidopsis B-box protein sequences (Table S3) were aligned by ClustalW using MEGA program [36].

### Microarray

Tissue was sampled from plants grown at Jerseyville, Illinois at R5 stage at 5 timepoints; ZT 21 (3am), ZT 0 (6am), ZT 3 (9am), ZT 6 (noon), and ZT 9 (3pm). Sunrise occurred at 6:08am, sunset at 8:08pm. As each collection timepoint required 15 minutes to sample, we set the sampling that initiated at 6am at ZT 0. Three bioreps pooled from three plants from each entry were collected. 200 mg ground plant tissue was aliquoted to a 2.0 ml lysing matrix E tube from Q-biogen. Nucleic acids were isolated by the CTAB method [37] and then precipitated overnight at −20°C in 800 µl 100 percent ethanol, 150 µl ammonium acetate and 3 µl glycogen. Pellets were washed 3× with 80 percent ethanol and resuspended in nuclease free water prior to DNase treatment for 1 h at 37°C. Total RNA was purified using the RNeasy kit from Qiagen according to the manufacturers' instructions. RNA yield was analyzed using a NanoDrop-1000 spectrophotometer and integrity by an Agilent 2100 Bioanalyzer. RNA amplification was performed according to the manufacturers' recommendations using the TargetAmp 1-round Biotin-aRNA amplification kit from EpiCentre. 12 µg of labeled RNA was then fragmented according to the standard protocols for gene expression analysis provided by Affymetrix. Fragmented cRNA samples were prepared and hybridized to custom GeneChips from Affymetrix according to the manufacturers' standard protocol.

Signal intensities were normalized using RMA (Robust Multi-Array Algorithm) using Partek software (St. Louis, MO) and subsequently transformed into log_2_ scale. A fixed effect ANOVA analysis was done on the log transformed data using the PROC MIXED module of SAS (V9.1.3) to identify genes having significant differential expression between the transgenic events and the wildtype control at each time point. Estimates of the fold change differential between the average response of the two transgenic events and the WT event were calculated by that module. The raw *p*-values for the estimated fold changes were adjusted to correct for the multiple testing problem using SAS's PROC MULTTEST module with the FDR method of Benjamini and Hochberg. Those genes with a FDR adjusted *p*-value less than 0.05 and an estimated differential fold change greater than 2.0 are reflected in Figure 2.

To identify genes with a significant temporal oscillation profile, the PROC NLIN module of SAS was used to fit the log_2_ transformed intensity values to a sine wave model:
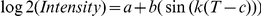
where *T* is the time point (24 hour scale) at which each sample is taken, *a*, *b*, and *c* are the fitted parameters computed by the NLIN model, and *k* is fixed at 2π/24 to constrain the model to a 24 hour cycle. Parameter *a* represents the average intensity across all the time points, *b* represents the magnitude of the diurnal response (2*b* is the peak to trough range in intensity), and *c* is the time shift for the sine wave (*c*+6 is the peak time and *c*−6 is the trough time). The raw *p*-values for the sine wave model were adjusted for multiple testing. Those genes with the FDR adjusted *p*-value less than 0.01 were selected and the list was further filtered to include only those where parameter *a* (average intensity) was above a background level and parameter *b* (diurnal magnitude) was greater than 0.5 so that there would be at least a two-fold change from peak to trough.

The microarray data discussed in this publication have been deposited in the NCBI Gene Expression Omnibus 39 and are accessible through GEO Series accession number GSE30828 (<http://www.ncbi.nlm.nih.gov/geo/query/acc.cgi?acc=GSE30828>).

### Quantigene RNA Extraction and Quantitative Expression Analysis

Analysis of diurnal profiles of selected transcripts was performed on *AtBBX32* events 1 and 2 from Table 1, *GmBBX52* event 7 from Table 4, *GmBBX53* event 2 from Table 4, and corresponding wild-type controls in a controlled environment growth chamber. Plants were grown with a 14 hour photoperiod at 28°C/22°C temperature (day/night), 60 percent humidity, and a fluence of 650 µE light. Plants were sampled beginning at 17 days after sowing (V2 stage). Six repetitions per entry consisting of 2 plants each were collected at each time point (ZT 23, ZT 1, ZT 2, ZT 5, ZT 8, ZT 11, ZT 14, ZT 17, and ZT 20). The first trifoliate leaves of V2 plants were collected and immediately frozen in liquid N_2_.

Tissue was milled and aliquoted to 96-well plates where RNA was extracted using the EZNA RNA Purification Kit (Omega BioTek, #r1027-02) following the manufacturer's standard protocols. RNA samples were then treated with Turbo DNA-Free DNase (Ambion). A QuantiGene Plex 2.0 Reagent System was designed and ordered (Panomics/Affymetrix Plex 41165). Upon receiving the assay panel, the assay plex was validated for assay precision (<15 percent CV), assay LOD/LOQ, assay linearity and accuracy of fold change. Based on the assay linearity and accuracy of fold change, the samples were normalized to 25 ng/ul and 0.5 ug was assayed. Median fluorescence intensity (MFI) values were obtained on a Luminex 200 instrument and Log2 transformed for statistical data analysis.

Statistical analyses were carried out using SAS 9.2 for Windows. An outlier analysis was performed first and outlier data points were discarded. Gene expression parameters were separately analyzed by a mixed effects model, fitting fixed effects for Entry, Timepoint and Timepoint *Entry, and fitting rep and rep*Event as random effects. The model can be described as below:
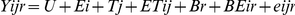
where Yijr = unique individual observation, U = overall mean, Ei = Event effect, Tj = Timepoint, ETij = Timepoint by Event interaction effect, Br = random effect of replicates, BEir = random replication by event interaction effect, eijr = experimental error.

### Transgene expression analysis

The transcript abundance of the transgenes and miRNA targets in this paper were assayed by Taqman analysis (Applied Biosystems). Sequence specific TaqMan assays were designed using Primer Express 2.0 software, where primers and probes (Table S5) were positioned at the polymorphic sites. Two TaqMan assays per target were run in duplex with internal control assay specific to 18S. All testing was done in ABI 7900HT real time cyclers. Assays detecting only the specific target, demonstrating efficiency of 90–110 percent and no endogenous control dependence were selected and tested for reproducibility. CV<2 percent was achieved at Log2 level over 3 log concentration variance, using synthetic controls.

## Supporting Information

Figure S1 **Phylogenetic analysis of the entire** ***A. thaliana*** **and** ***G. max*** **B-box gene family.**
(TIFF)

Figure S2 **The single B-box clade in** ***Arabidopsis thailana*** **and** ***Glycine max*** **.** The *Arabidopsis thaliana* genome contains seven single B-box domain genes while the paleopolyploid *Glycine max* genome contains thirteen single B-box genes. Phylogenetic analysis indicates that *GmBBX52* and *GmBBX53* are orthologs of the *Arabidopsis thaliana* BBX32 gene.
(TIFF)

Figure S3 **Overexpression of** ***GmBBX52*** **(line 7) or** ***GmBBX53*** **(line 2) in soybean affects the transcript abundance of central clock components near ZT 0.** Levels of both central clock components *GmLCL2* (A) and *GmTOC1* (B) were assayed by quantigene RNA extraction and expression analysis from V2 leaf tissue harvested from soybean plants grown in a controlled environment. Growth chamber experiment was performed in a 14:10 hour photoperiod (Light∶Dark) with 650 mE of light. p-values based on the difference between both transgenic lines and wildtype control. * p≤0.05. Where error bars are not visible they are smaller than the data points.
(TIFF)

Table S1 **Gene expression data for overexpression and miRNA targets described in this manuscript.**
(XLSX)

Table S2 **Commercial varieties used in this study.**
(XLSX)

Table S3 **BBX ID and the public gene IDs of the Arabidopsis and Soy Bbox genes described within the manuscript.**
(XLSX)

Table S4 **Changes observed in clock gene expression in the microarray experiment.**
(XLSX)

Table S5 **Primers used to quantitate transgene expression in this study.**
(XLSX)
