# Supplementary figures and images for: Expression of the Arabidopsis thaliana BBX32 Gene in Soybean Increases Grain Yield
Source: PLoS One. 2012 Feb 17;7(2):e30717. doi: 10.1371/journal.pone.0030717 (PMC3281879; doi:10.1371/journal.pone.0030717)

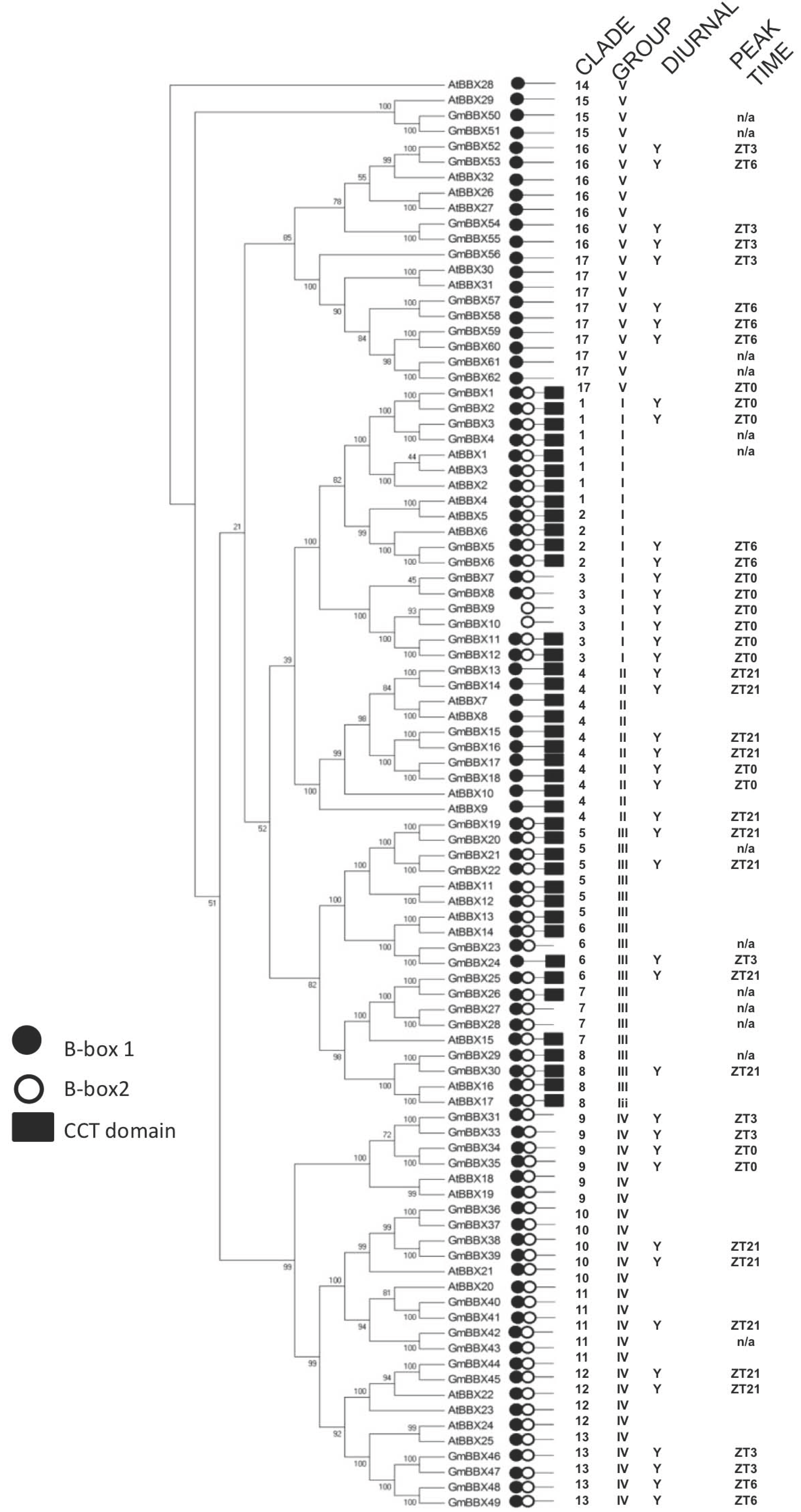

Supplement: Figure S1 — Phylogenetic analysis of the entire A. thaliana and G. max B-box gene family. (TIFF) [file pone.0030717.s001.tiff]

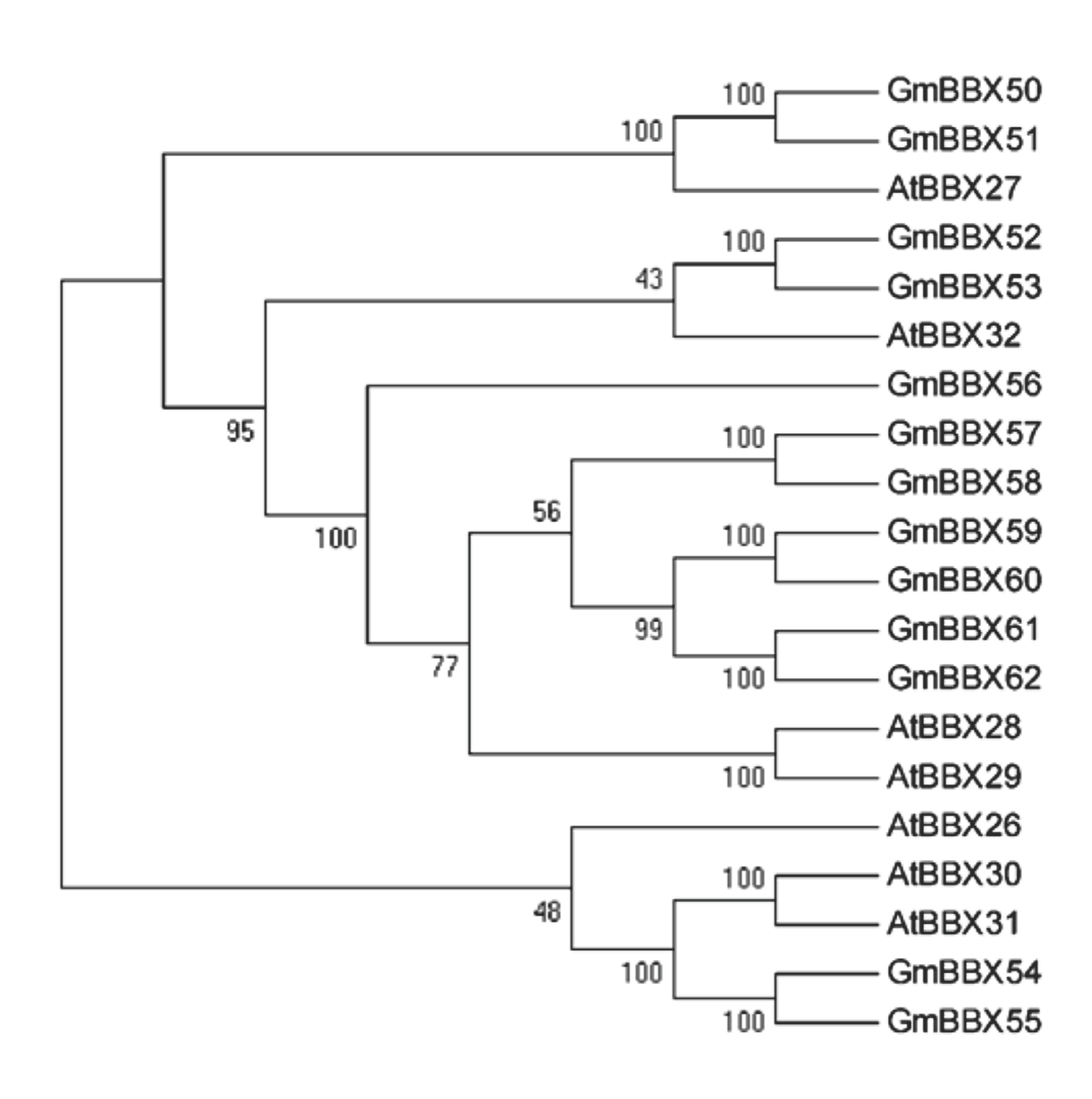

Supplement: Figure S2 — The single B-box clade in Arabidopsis thailana and Glycine max . The Arabidopsis thaliana genome contains seven single B-box domain genes while the paleopolyploid Glycine max genome contains thirteen single B-box genes. Phylogenetic analysis indicates that GmBBX52 and GmBBX53 are orthologs of the Arabidopsis thaliana BBX32 gene. (TIFF) [file pone.0030717.s002.tiff]

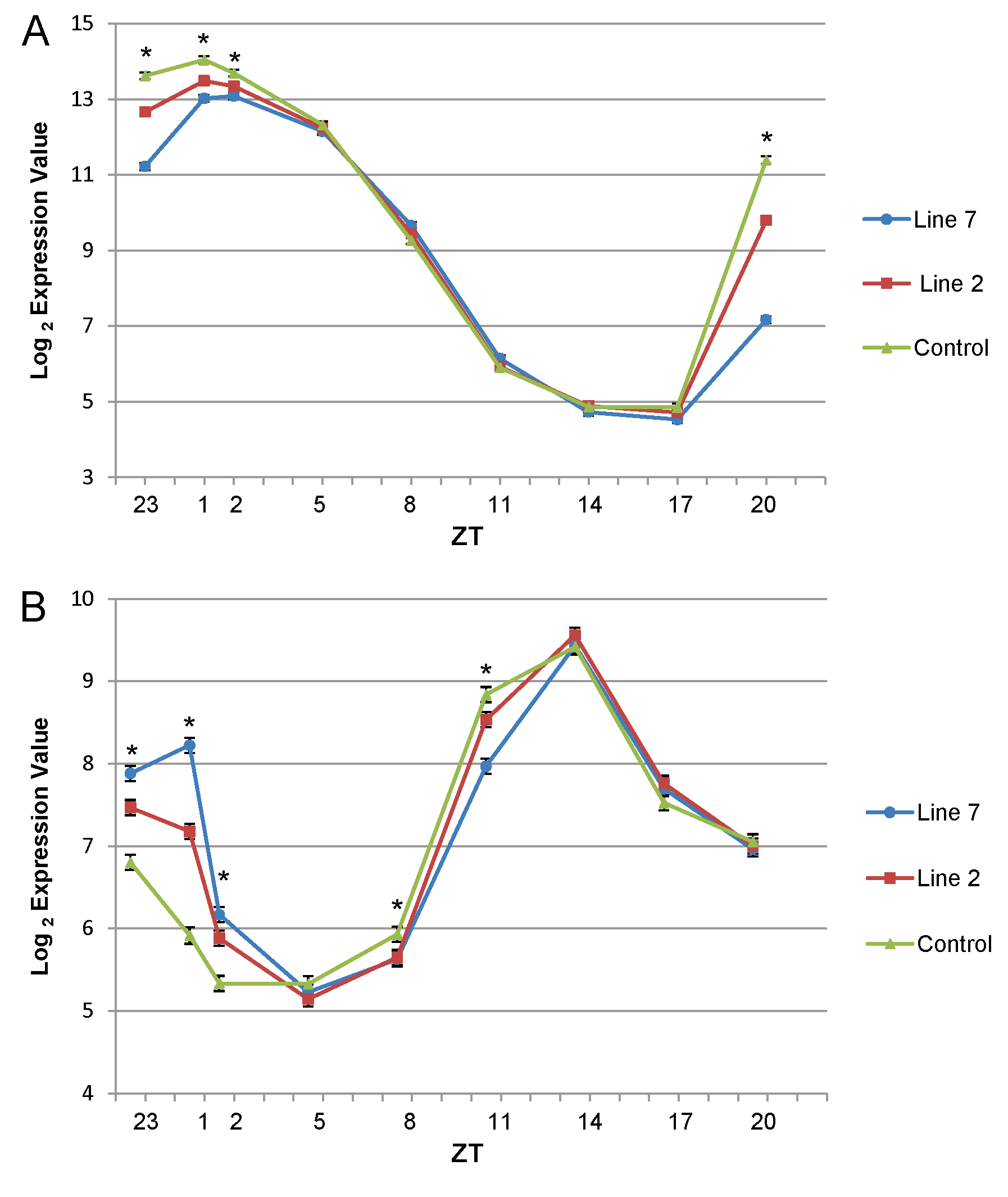

Supplement: Figure S3 — Overexpression of GmBBX52 (line 7) or GmBBX53 (line 2) in soybean affects the transcript abundance of central clock components near ZT 0. Levels of both central clock components GmLCL2 (A) and GmTOC1 (B) were assayed by quantigene RNA extraction and expression analysis from V2 leaf tissue harvested from soybean plants grown in a controlled environment. Growth chamber experiment was performed in a 14:10 hour photoperiod (Light∶Dark) with 650 mE of light. p-values based on the difference between both transgenic lines and wildtype control. * p≤0.05. Where error bars are not visible they are smaller than the data points. (TIFF) [file pone.0030717.s003.tiff]
